# Vitamin D and 1,25(OH)_2_D Regulation of T cells

**DOI:** 10.3390/nu7043011

**Published:** 2015-04-22

**Authors:** Margherita T. Cantorna, Lindsay Snyder, Yang-Ding Lin, Linlin Yang

**Affiliations:** 1 Department of Veterinary and Biomedical Science, The Pennsylvania State University, University Park, PA 16802, USA; E-Mails: lms496@psu.edu (L.S.); yxl254@psu.edu (Y.-D.L.); lyang1@hmc.psu.edu (L.Y.); 2 Center for Molecular Immunology and Infectious Disease, The Pennsylvania State University, University Park, PA 16802, USA

**Keywords:** vitamin D, T cells, vitamin D receptor

## Abstract

Vitamin D is a direct and indirect regulator of T cells. The mechanisms by which vitamin D directly regulates T cells are reviewed and new primary data on the effects of 1,25 dihydroxyvitamin D (1,25(OH)_2_D) on human invariant natural killer (iNK)T cells is presented. The *in vivo* effects of vitamin D on murine T cells include inhibition of T cell proliferation, inhibition of IFN-γ, IL-17 and induction of IL-4. Experiments in mice demonstrate that the effectiveness of 1,25(OH)_2_D requires NKT cells, IL-10, the IL-10R and IL-4. Comparisons of mouse and human T cells show that 1,25(OH)_2_D inhibits IL-17 and IFN-γ, and induces T regulatory cells and IL-4. IL-4 was induced by 1,25(OH)_2_D in mouse and human iNKT cells. Activation for 72h was required for optimal expression of the vitamin D receptor (VDR) in human and mouse T and iNKT cells. In addition, T cells are potential autocrine sources of 1,25(OH)_2_D but again only 48–72h after activation. Together the data support the late effects of vitamin D on diseases like inflammatory bowel disease and multiple sclerosis where reducing IL-17 and IFN-γ, while inducing IL-4 and IL-10, would be beneficial.

## 1. Introduction

Vitamin D is a fat soluble vitamin that is either consumed in the diet or produced in the skin following sunlight exposure of the skin. Vitamin D is inactive and is hydroxylated twice, once in the liver and once in the kidney to make the active form of the vitamin, 1,25 dihydroxyvitamin D (1,25(OH)_2_D) [1,2]. Production of 1,25(OH)_2_D in the kidney is tightly regulated by serum calcium, parathyroid hormone and 1,25(OH)_2_D levels [3]. Vitamin D as 1,25(OH)_2_D functions by binding to a nuclear vitamin D receptor (VDR) and retinoid X receptor to regulate gene transcription [4]. The classic roles of vitamin D are in the regulation of calcium uptake and homeostasis, bone metabolism, and cell growth and division. The VDR has been identified in many other tissues including the immune system and it is now accepted that 1,25(OH)_2_D and vitamin D are important immune system regulators [5]. All cells of the immune system have been shown to express the VDR including T cells [6].

## 2. T Cells

Several different types of T cells exist to provide defense from a variety of different insults to the body. CD4+ T cells provide help for B cell antibody production and to other cell types to engulf and kill pathogens. T helper (h) cell responses are heterogeneous although it has been shown that specific cytokine patterns are critical for control of susceptibility to infection. Th1 cells are important for the control of intracellular infections with *Mycobacterium tuberculosis*, *Listeria monocytogenes*, and a number of viruses [7,8,9]. Th2 cells that produce IL-4, IL-5, and IL-13 are required for host defense against parasitic helminthes [10]. Th17 cells produce IL-17, IL-22, and granulocyte macrophage-colony stimulating factor (GM-CSF) [11] and are essential for resistance to extracellular pathogens [12]. Additional T cells include the innate natural killer (NK)T cells. NKT cells recognize lipid antigens instead of protein antigens and produce large amounts of cytokines early during infection [13]. CD8+ T cells are cytotoxic T cells and are important for killing virally and bacterially infected cells. Each of these T cells are critical for immune protection from infection.

Dysregulation of T cell responses can cause pathology. Immune mediated diseases occur as a result of chronically activated T cells. T regulatory (reg) cells produce IL-10 and serve to inhibit effector T cell responses in an antigen specific and non-specific manner [14]. Deficiency of T reg cells results in multi-organ immune mediated disease [14]. Th1 and/or Th17 cells transfer experimental models of immune mediated diseases such as inflammatory bowel disease and multiple sclerosis. Experimental asthma and allergy models occur in animals that have Th2 cells, and in some cases NKT cells that overproduce IL-13 and/or IL-17 [15]. Control of the T cell response is therefore required for robust elimination of infection and the resolution of inflammation to prevent pathology due to chronic T cell activation.

## 3. Vitamin D and T Cells

The effects of vitamin D and 1,25(OH)_2_D as inhibitors of T cells have been well described. There are direct and indirect effects of 1,25(OH)_2_D on T cells but this review will focus on the direct effects. Since 1983 it has been described that 1,25(OH)_2_D inhibited T cell proliferation and the secretion of select cytokines after mitogen stimulation [16,17]. Moreover, 1,25(OH)_2_D directly inhibited IL-2 and IFN-γ transcription [17,18]. More recently 1,25(OH)_2_D has also been shown to inhibit IL-17 secretion by Th17 cells [19,20]. The effects of 1,25(OH)_2_D on Th2 cells is more controversial with evidence that 1,25(OH)_2_D inhibits IL-4 transcriptionally as well as evidence that 1,25(OH)_2_D upregulates IL-4 in mouse and human T cells [20,21,22,23]. *In vitro*, 1,25(OH)_2_D3 treatments induced IL-10 and T regulatory cell development [24]. In addition, 1,25(OH)_2_D upregulated the gut homing receptor CCR9 and inhibited CXCR3 on T cells potentially changing the homing properties of the Th cells [25]. Vitamin D and 1,25(OH)_2_D inhibited Th1 and Th17 responses, induced T reg responses, and controlled proliferation and Th cell localization.

CD8+ T cells and iNKT cells are also vitamin D targets. *In vitro*, 1,25(OH)_2_D treatment inhibited CD8 T cell proliferation and VDR knockout (KO) CD8+ T cells proliferated without antigen stimulation due in part to over-production of IL-2 [26,27]. VDR KO CD8 T cells had altered homing patterns, a reduction in granzyme B production and more rapid contraction in an infection model [28]. Other defects in VDR KO CD8+ T cells included the ability, when transferred to leucopenic hosts, to develop into IL-17 and IFN-γ secreting cells that produced colitis symptoms [26]. In the gastrointestinal tract, CD8αα/TCRαβ T cells important in maintaining homeostasis, required vitamin D to maintain their homeostatic proliferation [29]. Development and function of iNKT cells depends on expression of the VDR and vitamin D [30,31]. *In vitro*, 1,25(OH)_2_D inhibited iNKT cell derived IL-17 and induced IL-4 and IL-5 [32]. The requirement for the VDR in the development of iNKT cells was traced to regulation of the survival of maturing iNKT cells in the thymus [31]. Vitamin D controls CD8 proliferation, IL-2 production and the potential to develop into effector T cells that produce IFN-γ, IL-17 and granzyme B. Vitamin D also controls iNKT cell expansion during development. Lastly vitamin D changes early cytokine production by iNKT cells that could shape later T cell responses.

The effectiveness of vitamin D and 1,25(OH)_2_D treatments on animal models of T cell mediated diseases has been informative. Th1 and Th17 cells cause experimental autoimmune encephalomyelitis (EAE, murine model of multiple sclerosis), inflammatory bowel disease and type-1 diabetes. *In vivo*, 1,25(OH)_2_D treatments suppressed the development and progression of these Th1/Th17 mediated diseases [33,34,35]. In addition, vitamin D and VDR deficiency exacerbated experimental type-1 diabetes and inflammatory bowel disease in mice [33,35,36]. In EAE the effectiveness of 1,25(OH)_2_D has been shown to require iNKT cells, IL-4, IL-10, and L-10R [32,37,38]. In addition, the 1,25(OH)_2_D-mediated inhibition of IL-17 and IFN-γ with the induction of IL-10 and T reg cells have been suggested as mechanisms to explain suppression of experimental EAE, IBD and diabetes [24,39,40,41].

Vitamin D has also been proposed as a regulator of Th2 mediated disease such as allergy and asthma. *In vitro*, 1,25(OH)_2_D treatment of T cells has been shown to increase IL-4 secretion by human and mouse Th cells [21,42,43]. IL-13 has been shown to be induced [44] or decreased [45] by 1,25(OH)_2_D treatment of human T cells. VDR KO Th2 cells made less IL-4 on the C57BL/6 background and less IL-4, IL-5 and IL-13 on the Balb/c background [46]. In addition, VDR KO mice on either the Balb/c or C57BL/6 background failed to develop experimental allergic asthma [46]. However, cell transfer and bone marrow transplantation showed that VDR KO T cells could induce asthma in the WT host and therefore VDR expression in the lung might account for the increased resistance of the VDR KO mice to allergic asthma [47]. iNKT cells also contribute to asthma development and 1,25(OH)_2_D induced IL-4, and IL-5 from iNKT cells [32,48]. The data on the effects of 1,25(OH)_2_D treatments of experimental allergic asthma show contradictory results with no effects, worsening of symptoms and symptom amelioration [49,50,51]. The effects of 1,25(OH)_2_D on T reg cells and IL-10, that also suppress the Th2 response, could explain some of the beneficial effects of vitamin D in experimental asthma [52,53,54]. Overall the data support that vitamin D and 1,25(OH)_2_D induce production of Th2 cytokines. Other vitamin D targets in the lung, T reg cells and/or other immune cells are likely the explanation for the contradictory results in the experimental allergic asthma models.

## 4. Vitamin D Regulation of Mouse *versus* Human T Cells

Much of the work describing the basic mechanisms of vitamin D, the VDR and 1,25(OH)_2_D on T cells *in vivo* have been done in mice. These *in vivo* experiments are difficult to replicate in humans. However, since the goal is to use mice to model the effects of vitamin D and 1,25(OH)_2_D in humans, it is important to determine which of the effects of vitamin D in murine T cells can also be observed in human T cells. It should be noted however, that much of the work using human T cells is done with peripheral blood mononuclear cells (PBMC). In the mouse the T cells studied come from different tissues (usually not the blood) and the functions of the T cells depend to a large extent on where they are located.

The early work utilized human PBMC to demonstrate that T cells expressed the VDR and were vitamin D targets. Human CD8 and CD4 T cells were inhibited from proliferating in the presence of 1,25(OH)_2_D [18,27,55]. In addition, 1,25(OH)_2_D inhibited IL-2, IFN-γ, and IL-17 in human and mouse T cells [21,42,43,56]. Freshly isolated PBMC were stimulated with CD3 and CD28 antibodies or αGalCer in the presence of 0-50nM 1,25(OH)_2_D. Confirming the literature, our experiments also showed 1,25(OH)_2_D inhibited IFN-γ and T cell proliferation and induced IL-4 production from PBMC stimulated with CD3/CD28 (data not shown). Activation of both human and mouse T cells induced expression of the VDR and it took 48-72h to induce VDR protein in the T cells [6,21,57]. Human Th1, Th2 and Th17 cells expressed similar and high amounts of the VDR protein 72 hours after activation [57]. The amount of 1,25(OH)_2_D addition to activated T cells protected the VDR protein from proteasomal degradation and 1,25(OH)_2_D has been shown to stabilize VDR protein in other cell types as well [57,58]. In addition, activation of mouse CD8+ T cells and human CD4+ T cells for 48-72 hours induced expression of the vitamin D 1-alpha hydroxylase (Cyp27B1) suggesting that T cells might be able to locally produce 1,25(OH)_2_D [59,60]. In human PBMC 1,25(OH)_2_D induced the expression of IL-4 when added *in vitro* and 1,25(OH)_2_D induced human T reg development and IL-10 production [42,43,61]. Collectively the effects of vitamin D, production of Cyp27B1 and 1,25(OH)_2_D on mouse T cells *in vitro* reflect the effects of 1,25(OH)_2_D on human T cells from the PBMC.

**Figure 1:**
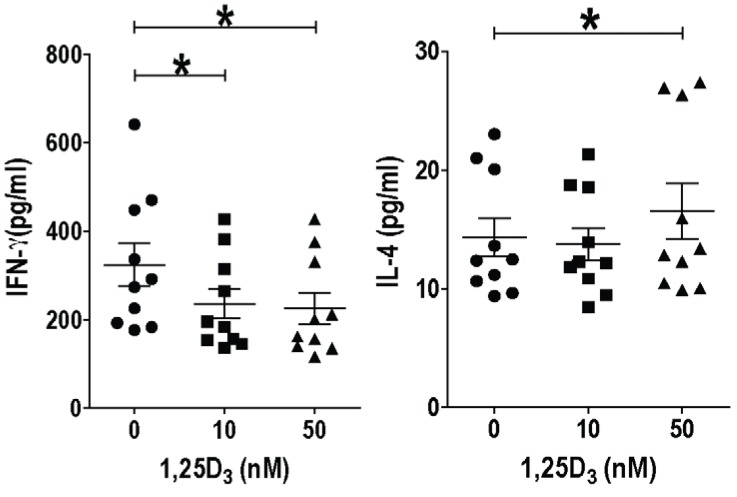
Fresh peripheral blood mononuclear cells (PBMCs) stimulated with α-Galcer were cultured for 72 h with or without 1,25(OH)_2_D treatment and supernatants were analyzed by ELISA for IFN-γ and IL-4 production. *n* = 10 different PBMC donors. * *P* < 0.05. Experiments using humans were done with the approval of the Pennsylvania State University, University Park, PA, Institutional Review Board: #32141.

**Figure 2:**
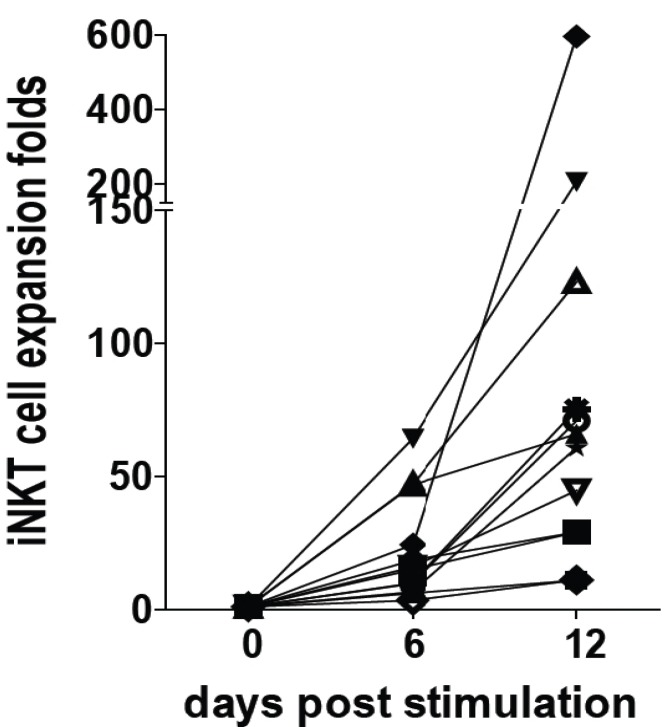
iNKT cell expansion *ex vivo*. PBMCs were stimulated with 100 ng/mL α-GalCer for 12 days to expand iNKT cells. *n* = 12 individual donors were used.

**Figure 3:**
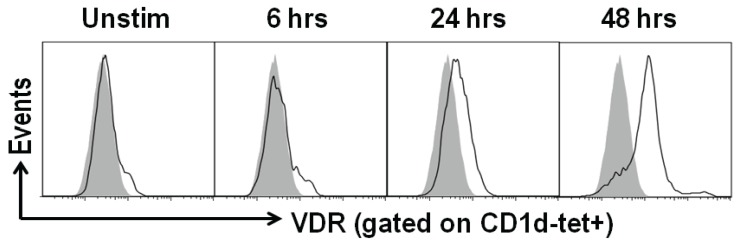
Vitamin D receptor (VDR) expression is up regulated with activation. iNKT cell lines were restimulated with α-GalCer and pulsed irradiated PBMCs *in vitro*. Cells were harvested at 6, 24 and 48 h post stimulation. iNKT cells (gated on CD1d-tet+ cells) were stained with anti-VDR antibodies (Clone H4537, R&D Systems) The isotype control staining is the grey histogram. Data shown is one representative of two experiments performed done with iNKT cell lines from 2 different donors.

PBMC are readily accessible sources of human immune cells including iNKT cells. However, the frequencies of iNKT cells (CD1d tetramer+/CD3+) in the PBMC is very low and ranged from 0.008%–0.292% of the cells (data not shown). α-Galactoceramide (GalCer), an iNKT cell specific ligand, was used to stimulate freshly isolated human PBMC. IFN-γ was inhibited by 10 and 50 nM of 1,25(OH)_2_D addition to αGalCer stimulated PBMC (Figure 1). IL-4 went up with 50nM but not 10 nM 1,25(OH)_2_D (Figure 1). Cultures were set up to expand and purify the iNKT cells. The ability to expand iNKT cells from some donors was low (11–29 fold expansion) while iNKT cell expansion from 3 individuals was high (123–596 fold expansion, Figure 2). Adding 10 and 50 nM 1,25(OH)_2_D at the start of the 12 day culture reduced the iNKT cells that could be recovered from the cultures (data not shown). Cell lines were generated utilizing magnetic bead purified iNKT cells (95% CD1d tetramer+/CD3+) for experiments. Resting iNKT cell lines had very low expression of the VDR that was upregulated with 48h of αGalCer and irradiated PBMC incubations (Figure 3). There was not an effect of 1,25(OH)_2_D on the proliferation of three different human iNKT cell lines (data not shown). The data we provide here suggest that there are important differences between the effects of 1,25(OH)_2_D on tissue iNKT cells in the mouse and PBMC iNKT cells in the human. Human iNKT cells, like T cells, optimally express the VDR after several days of activation. Induction of IL-4 by 1,25(OH)_2_D in murine and human iNKT cells was the same. The reason for the difference between the effects of 1,25(OH)_2_D on mouse and human iNKT cells might be a species difference, the location of the cells, the low frequencies of the iNKT cells in the freshly isolated PBMC or changes that occur *in vitro* when culturing and expanding human iNKT cells.

## 5. Conclusions

T cells become vitamin D targets by expressing the VDR and inducing autocrine 1,25(OH)_2_D following activation. The 1,25(OH)_2_D inhibits murine and human T cells from proliferating, producing IFN-γ, and IL-17 while inducing IL-4. In animal models these effects correspond to the vitamin D mediated inhibition of experimental immune mediated diseases caused by Th1 and Th17 cells. Further, 1,25(OH)_2_D induced IL-4 in both mouse and human iNKT cells. The effects of 1,25(OH)_2_D on proliferation and IFN-γ production in mouse and human iNKT cells was different. Overall the effects of vitamin D on both iNKT cells and T cells seem to require 48–72 h of activation and suggest that vitamin D is important late during the T cell response. Together the data suggest an important role for vitamin D and 1,25(OH)_2_D in regulating T cells to limit immune mediated diseases where IL-17 and IFN-γ, are pathogenic.
